# Engineering of the glycerol decomposition pathway and cofactor regulation in an industrial yeast improves ethanol production

**DOI:** 10.1007/s10295-013-1311-5

**Published:** 2013-07-30

**Authors:** Liang Zhang, Yan Tang, Zhongpeng Guo, Guiyang Shi

**Affiliations:** 1 The Key Laboratory of Industrial Biotechnology, Ministry of Education, Jiangnan University, Wuxi, People’s Republic of China; 2 National Engineering Laboratory for Cereal Fermentation Technology of Jiangnan University, Wuxi, People’s Republic of China; 3 Department of Chemical and Biological Engineering, Industrial Biotechnology, Chalmers University of Technology, Gothenburg, Sweden

**Keywords:** Industrial yeast, Glycerol, Ethanol production, Cofactor NADH, Recombinant strain

## Abstract

Glycerol is a major by-product of industrial ethanol production and its formation consumes up to 4 % of the sugar substrate. This study modified the glycerol decomposition pathway of an industrial strain of *Saccharomyces cerevisiae* to optimize the consumption of substrate and yield of ethanol. This study is the first to couple glycerol degradation with ethanol formation, to the best of our knowledge. The recombinant strain overexpressing *GCY1* and *DAK1*, encoding glycerol dehydrogenase and dihydroxyacetone kinase, respectively, in glycerol degradation pathway, exhibited a moderate increase in ethanol yield (2.9 %) and decrease in glycerol yield (24.9 %) compared to the wild type with the initial glucose concentration of 15 % under anaerobic conditions. However, when the *mhpF* gene, encoding acetylating NAD^+^-dependent acetaldehyde dehydrogenase from *Escherichia coli*, was co-expressed in the aforementioned recombinant strain, a further increase in ethanol yield by 5.5 % and decrease in glycerol yield by 48 % were observed for the resultant recombinant strain GDMS1 when acetic acid was added into the medium prior to inoculation compared to the wild type. The process outlined in this study which enhances glycerol consumption and cofactor regulation in an industrial yeast is a promising metabolic engineering strategy to increase ethanol production by reducing the formation of glycerol.

**Electronic supplementary material:**

The online version of this article (doi:10.1007/s10295-013-1311-5) contains supplementary material, which is available to authorized users.

## Introduction

In industrial ethanol fermentation, glycerol is a major by-product whose production can consume up to 4 % of the carbon source [17]. If the carbon flow towards glycerol production can be redirected towards ethanol synthesis, the yield of ethanol and efficiency of raw material use can be improved. In the commercially used yeast *Saccharomyces cerevisiae*, glycerol is synthesized in a two-step reaction catalyzed by NAD^+^-dependent glycerol-3-phosphate dehydrogenase (GPD) and glycerol-3-phosphate phosphatase (GPP). GPD is encoded by two highly homologous isogenes *GPD1* and *GPD2*, and is rate-controlling in the formation of glycerol [7].

Glycerol synthesis plays a significant physiological role in the metabolism of yeast by protecting yeast cells from osmotic stress [4, 16] and maintaining a redox balance by converting surplus NADH to NAD^+^ under anaerobic conditions [1, 21]. In addition, glycerol can be converted to glycolytic intermediates via the glycerol-3-phosphate route under aerobic conditions and by the dihydroxyacetone route under microaerobic conditions in *S. cerevisiae*. The dihydroxyacetone pathway involves a glycerol dehydrogenase encoded by *GCY1* or *YPR1*, and a dihydroxyacetone kinase encoded by *DAK1* or *DAK2* [6]; however, the physiological role of this pathway has not been elucidated.

Various attempts have been made to improve ethanol production by reducing glycerol formation. In one study, interruption of glycerol production by deletion of *GPD1* or *GPD2*, or both, proved unsuccessful because the growth rate and by-product formation in such engineered strains were severely curtailed [9].

Other strategies to reduce glycerol production have involved cofactor regulation in which NADH formation has been curtailed. For example, when the cofactor specificity of glutamate dehydrogenase was altered to increase NADH consumption in *S. cerevisiae*, ethanol yield increased by 8 % [17]. However, this process required sufficient biomass to reach completion at a satisfactory level. In a separate study, a non-phosphorylating NADP^+^-dependent glyceraldehyde-3-phosphate dehydrogenase from *Streptococcus* mutants was expressed to replace NAD^+^-dependent glyceraldehyde-3-phosphate dehydrogenase in *S. cerevisiae*; as a result, ethanol production increased by 24 % while glycerol yield decreased by 58 % compared to the reference strain [5]. Another novel metabolic engineering strategy involved the expression of a NAD^+^-dependent acetylating acetaldehyde dehydrogenase from *Escherichia coli* in a *gpd1*△ *gpd2*△ strain of *S. cerevisiae*, thus using a linear pathway for the NADH-dependent reduction of acetic acid to ethanol to replace glycerol formation as a redox sink in anaerobic ethanol production. The *GPD1* △*GPD2*△ *S. cerevisiae* expressing the *E. coli mhpF* gene was able to grow under anaerobic conditions when the media was supplemented with 2.0 g/l acetic acid, and the ethanol yield increased by 13 %. However, growth and product formation were significantly slower in that engineered strain [14]. Furthermore, when NADH kinase was overexpressed in *S. cerevisiae* to catalyze the conversion of NADH into NADPH, the carbon dioxide (CO_2_) was converted to ethanol and acetate during anaerobic growth on glucose [10].

In our previous study, three different genes were overexpressed in an industrial yeast strain separately: *gapN*, encoding a non-phosphorylating NADP^+^-dependent glyceraldehyde-3-phosphate dehydrogenase in *Bacillus cereus*; *frdA*, encoding the NAD^+^-dependent fumarate reductase; and *mhpF*, encoding the acetylating NAD^+^-dependent acetaldehyde dehydrogenase in *E. coli*. Overexpression of *mhpF* in *S. cerevisiae* generated the best improvement in ethanol yield by 4.3 % and greatest decrease in glycerol yield by 40 % compared to the wild type when acetic acid was added prior to inoculation [24].

**Fig. 1:**
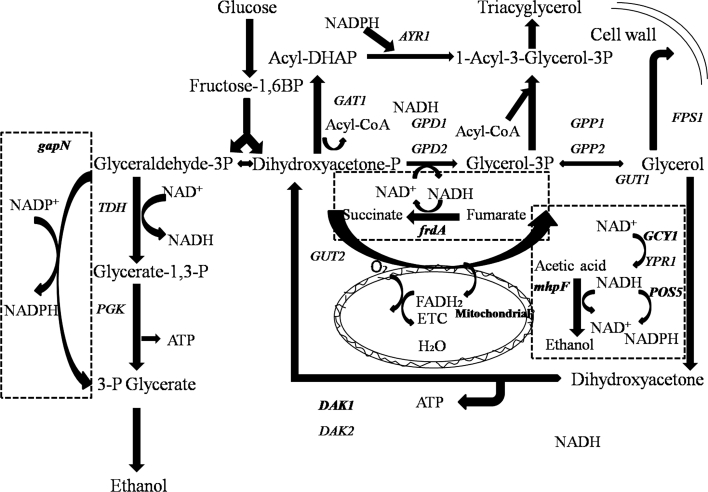
Ethanol and glycerol metabolism in *Saccharomyces cerevisiae*: *GCY1* encoding glycerol dehydrogenase; *DAK1* encoding dihydroxyacetone kinase; *gapN* encoding the non-phosphorylating NADP^+^-dependent glyceraldehyde-3-phosphate dehydrogenase; *mhpF* encoding the acetylating NAD^+^-dependent acetaldehyde dehydrogenase; *frdA* encoding the NAD^+^-dependent fumarate reductase; *POS5* encoding the NADH kinase. The four introduced genes and corresponding reactions are shown in the *dashed boxes*

In this study, we investigated the effects of overexpressing the genes *GCY1* and *DAK1* in *S. cerevisiae* on anaerobic glycerol degradation to dihydroxyacetone phosphate which was then converted to ethanol (Fig. 1). The *S. cerevisiae* *POS5* gene encoding NADH kinase and *E. coli* *mhpF* gene were overexpressed in the recombinant yeast in which *GCY1* and *DAK1* were introduced, respectively. The aim was to evaluate whether modification of the glycerol decomposition pathway and cofactor regulation could improve ethanol production. By overexpressing different genes controlling NADH production, and comparing the results with those from previously constructed strains, the most promising strategies of cofactor regulation to increase ethanol yield and decrease glycerol production were determined in *S. cerevisiae.*

## Materials and methods

### Yeast strains and media

The strains and plasmids used in the present study are summarized in Table 1. *E. coli* was grown in LB medium containing 5 g/l yeast extract, 10 g/l Bacto Peptone, and 10 g/l NaCl. *S. cerevisiae* used for genetic manipulation was routinely cultivated in YEPD medium containing 10 g/l yeast extract, 20 g/l Bacto Peptone, and 20 g/l glucose. Solid media contained 2 % agar. For yeast transformation, G418 or Hygromycin B was added to a final concentration of 500 or 250 μg/ml, respectively. Incubation conditions were standardized on a rotary shaker at 30 °C and 150 rpm.

**Table 1:** Strains and plasmids used in the present study

Strains/plasmids	Relevant genotype	Source of reference
Strains		
*Saccharomyces cerevisiae*	Polyploid, wild type	CICIMY0086, JU
*Escherichia coli* JM109	recA1 supE44 endA1 hsdR17 (rK^−^, mK^+^) gyrA96 relA1 thi (lac-proAB)F’[traD36 proAB^+^ lacI^q^ lacZM15]	CICIM-CU
*S. cerevisiae* GAS1	Polyploid,∷P_TP1_-*gapN*-*kan* ^*r*^	Zhang et al. [24]
*S. cerevisiae* MHS1	Polyploid,∷P_TP1_-*mhpF*-*kan* ^*r*^	Zhang et al. [24]
*S. cerevisiae* FRS1	Polyploid,∷P_TP1_-*frdA*-*kan* ^*r*^	Zhang et al. [24]
*S. cerevisiae* GDS1	P_PGK_-*GCY1*-*DAK1*- *kan* ^*r*^	This study
*S. cerevisiae* POS1	Polyploid,∷P_TP1_-*POS5*-*kan* ^r^	This study
*S. cerevisiae* GDMS1	P_PGK_-*GCY1*-*DAK1* P_TP1_–*mhpF*-*kan* ^*r*^	This study
Plasmids		
pYX212-*kan*-*POS5*	*kan* TP1_PT_-*POS5*	This study
pSH47-*HyBR*	*HyBR*	Guo et al. [9]
pMGKR	*kan* PGK1_PT_	This study
pMGKR-*GCY1*	*kan* PGK1_PT_-*GCY1*	This study
pMGKR-*DAK1*	*kan* PGK1_PT_-*DAK1*	This study
pMGKR-*GCY1*-*DAK1*	*kan* PGK1_PT_-*GCY1*-*DAK1*	This study

### Construction of strains

#### Expression of *GCY1* and *DAK1*

Details of the primers used in this study are listed in Table 2. The plasmid used for expression of *GCY1* and *DAK1* was constructed as follows: the PGK1 promoter and terminator were cloned by PCR amplification from *S. cerevisiae* genomic DNA with primers PF1 and PR2, TF1 and TR2, respectively. The two fragments obtained were digested by *Sal*I, and then inserted into pMD18-T simple vector to create plasmid pMG. The gene which confers resistance to geneticin (G418) in *S. cerevisiae* was isolated from pPIC9K using primers KF1 and KR2. The primers used to amplify this fragment were designed to introduce the 34-bp *loxp* site at the 5′- and 3′-ends. The PCR product was cut with *Not*I and inserted into the corresponding site of pMG. Following this, a partial rDNA fragment of *S. cerevisiae* used as a homologous integration site was cloned with primers RF1 and RR2, and ligated into the* Nde*I site of pMG, forming pMGKR. *GCY1* and *DAK1* were amplified from *S. cerevisiae* genomic DNA with primers GCY1F and GCY1R, DAK1F and DAK1R, respectively. The amplified fragments were digested by *Eco*RI and *Sal*I, and inserted into the relevant site of pMGKR, resulting in pMGKR-*GCY1* and pMGKR-*DAK1*, respectively. The PGK1_PT_-*DAK1* gene cassette was then amplified from pMGKR-*DAK1* with primers PF1 and TR2, and then cloned into a unique *Kpn*I site of pMGKR-*GCY1* to create pMGKR-*GCY1*-*DAK1* (supplementary Fig. 1). The plasmid pMGKR-*GCY1*-*DAK1* was linearized with *Sac*II. Following purification, the resultant linear DNA fragment was introduced into *S. cerevisiae* CICIMY0086 via the lithium acetate method. G418 was added to a final concentration of 0.5 mg/ml for yeast selection. The recombinant strain of *S. cerevisiae* was designated GDS1 (P_PGK_-*GCY1*-*DAK1*).

**Table 2:** Primers used in the present study

Primer name	Sequence (5′–3′)^a^	Restriction sites
PF1	CCGAAGCTTATTTTAGATTCCTGACTTCAACTC	*Hin*dIII
PR2	GCGGAATTC GTCGACTTCTTTGGAATTATTGGAAGGTA	*Eco*RI*, Bam*HI
TF1	CCGGAATTC GTCGACTTCTTTGGAATTATTGGAAGGTA	*Eco*RI*, Sal*I
TR2	GCGGAGCTC GGTACCGAACGCAGAATTTTCGAGTTAT	*Sac*I*, Kpn*I
RF1	CCGCATATGCTCTATCCCCAGCACGA	*Nde*I
RR2	CCCCATATGGAGAAACGGCTACCACATC	*Nde*I
KF1	AGGCGGCCGC ATAACTTCGTATAATGTATGCTATA CGAAGTTATGCCCAGTAGTAGGTTGAGG	*NotI*, *loxp*
KR2	AGGCGGCCGC ATAACTTCGTATAATGTATGCTATA CGAAGTTATTTGAAGTCGGACAGTGAGT	*NotI*, *loxp*
KMF	AGGGTACCGCCCAGTAGTAGGTTGAGG	*KpnI*
KMR	AGGGTACCTTGAAGTCGGACAGTGAGT	*KpnI*
GCY1F	CCGGAATTCATGCCTGCTACTTTACATGATTCT	*Eco*RI
GCY1R	CGCGTCGACTTACTTGAATACTTCGAAAGGAG	*Sal*I
DAK1F	CCGGAATTCATGTCCGCTAAATCGTTTGAAGTC	*Eco*RI
DAK1R	CGCGTCGACTTACAAGGCGCTTTGAACCCCCTT	*Sal*I
POS5F	CGCGGATCCATGAGTACGTTGGATTCACATTC	*Bam*HI
POS5R	CGCGTCGACTTAATCATTATCAGTCTGTCTCTTG	*Sal*I
HyrF	GGGGGTACCACATTTTGATGGCCGCACGG	*Kpn*I
HyrR	GGGGGTACCAACTCCTTCCTTTTCGGTTAGAGCG	*Kpn*I

^a^Restriction sites are underlined

#### Expression of *POS5*

The kanamycin resistance gene was cloned into the *Kpn*I site of pYX212 (Ingenius MBV-028-10) using the pPIC9K vector as a template by PCR with primers KMF and KMR, yielding pYX212-*km*. The *POS5* gene was amplified from *S. cerevisiae* genomic DNA by PCR using primers POS5F and POS5R containing *BamH*I and *Sal*I sites on each 5′-end. The gene was digested by *BamH*I and *Sal*I, and then inserted into the same sites of pYX212-*km* to form pYX212-*POS5*-*km*. The recombinant plasmids obtained were introduced into *S. cerevisiae* CICIMY0086 via the lithium acetate method [11].

#### Expression of *mhpF* in recombinant strain *S. cerevisiae* GDS1

The Cre recombinase expression vector pSH47 was introduced into the recombinant strain GDS1 and the G418 resistance gene of GDS1 was deleted using the *Cre/loxp* system [8]. Following this, the *mhpF* gene was expressed in the resultant recombinant strain *S. cerevisiae* GDS1 (P_PGK_-*GCY1*-*DAK1*).

### Enzyme assays

#### Measurement of GCY1 and DAK1 activity

The activity of glycerol dehydrogenase and dihydroxyacetone kinase was measured according to the method described previously with slight modifications [19]. The activity of glycerol dehydrogenase was measured in a reaction mixture (1 ml) containing 2 mM MgCl_2_, 500 mM NADH, 100 mM hydroxyacetone, 30 μl crude cell extract, and 100 mM of the appropriate buffer according to the pH of the assay. The activity of dihydroxyacetone kinase was recorded as the amount of NADH oxidized per unit of time in a coupled reaction with excess glycerol-3-phosphate dehydrogenase, where the reaction was started by adding 4 mM DHA [15]. One unit of the overall glycerol dehydrogenase and dihydroxyacetone kinase activity was defined as the amount of enzyme required to produce 1 μmol of NAD^+^ per minute from the NADH.

#### Measurement of POS5 activity

The recombinant strains were cultured for 72 h at 30 °C and the cells were collected by centrifugation at 8,000*g* for 5 min. After being washed twice with potassium phosphate buffer (PBS, 100 mmol/l, pH 7.4), the yeast cells were resuspended with the same buffer, and disrupted using a sonic dismembrator (VC750, Sonics, USA) at 30 % of the total working energy for 5 min at 0 °C to determine the enzyme activity. NADH kinase activity was measured spectrophotometrically at 340 nm according to the procedure described previously [20]. One unit of the NADH kinase activity was defined as the amount of enzyme required to produce 1 μmol of NADPH per minute from the NADH.

#### Measurement of the intracellular NADH concentration

Three replicate samples obtained after 12 h of fermentation were used to measure the intracellular NADH concentration. A 3-ml cell suspension was immediately added to 1.0 ml of 1 M alcoholic KOH (−20 °C, 50 % v/v ethanol) and an equal volume of glass beads (D 0.5 mm) to extract NADH. Under these conditions, the concentrations of NADH remained fairly constant for a period of 2 h. The optimal method to extract nucleotides was to alternate between oscillation and freezing over eight cycles, after which the mixture was incubated for 7 min at 70 °C [12] to maximize the recovery of reduced pyridine nucleotides. After cooling to 0 °C, the samples were adjusted to pH 7.0 by careful addition of 0.5 M HCl and thorough vortexing. The extracts were then processed in an enzymatic cycling system [13].

### Cultivation conditions

Yeasts were pre-cultured in 500-ml Erlenmeyer flasks at 30 °C in YEPD medium until an OD_600_ value of 10 was achieved (approximately 29 × 10^8 ^cells/ml). This pre-culture was used to inoculate the fermentation medium to yield an initial OD_600_ of 0.4 (0.30 mg/ml dry mass). In order to maintain anaerobic conditions in batch fermentations, the flasks were stoppered with a rubber bung into which a vent-pipe had been placed. The medium for fermentation contained 150 g/l glucose supplemented with 7.5 g (NH_4_)_2_SO_4_, 3.5 g KH_2_PO_4_, 0.75 g MgSO_4_·7H_2_O, and 0.5 g yeast extract, 420 mg Tween 80, and 10 mg ergosterol [22]. Aliquots of 300 μl/l antifoam were added to prevent foaming. During the fermentation process, the flasks containing 150 ml medium were kept at 30 °C in a thermostatic chamber with magnetic stirring. Fermentation experiments were performed in triplicate.

### Analysis of product formation and determination of dry weight

The concentrations of glucose, ethanol, and glycerol in filtered samples withdrawn from the batch cultivations were determined by high-performance liquid chromatography (HPLC) using an SH1011 column (Agilent, USA) and eluted with 0.01 M H_2_SO_4_ at 50 °C. Biomass was measured gravimetrically as described earlier [18]. The product yield was calculated as the ratio of product obtained to substrate consumed at the end of fermentation.

## Results

### Construction of recombinant yeast strains* S. cerevisiae* GDS1, POS1, GDMS1

Successful transformation of the linear DNA for overexpression of *GCY*1 and *DAK*1, and the recombinant plasmid for overexpression of *POS*5 in yeast cells were verified by PCR. The enzymatic activities of glycerol dehydrogenase, dihydroxyacetone kinase, NADH kinase, and NAD^+^-dependent acetaldehyde dehydrogenase of 20 colonies of the transformants were investigated according to the method previously described [15, 20]. Three recombinant strains denoted GDS1 (P_PGK_-*GCY1*-*DAK1*), POS1 (P_TP1_-*POS5*-*kan* ^*r*^), and GDMS1 (P_PGK_-*GCY1*-*DAK1,* P_TP1_-*mhpF*-*kan* ^*r*^) showed the highest activities for recombinant enzymes (1.3 IU/mg protein, 2.1 IU/mg protein, 3.98 IU/mg protein, respectively) compared to the parent strain; these were used in subsequent experiments (IU, the amount of enzyme required to convert 1 μmol of product per minute from the substrate, 1 IU = 1 μmol/min).

**Fig. 2:**
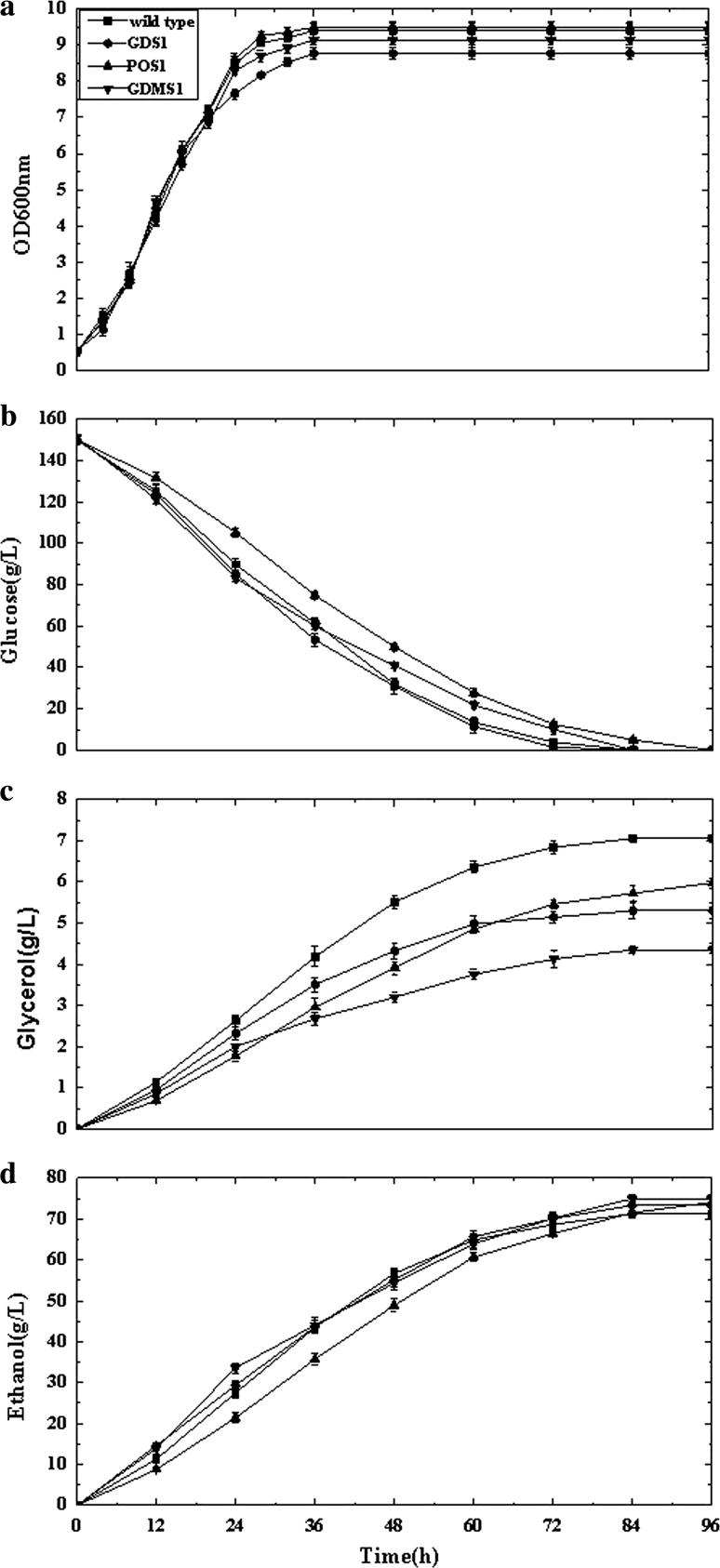
Comparison of recombinant strain *S. cerevisiae* GDS1, *S. cerevisiae* POS1, *S. cerevisiae* GDMS1, and wild type: **a** OD_600_, optical density at 600 nm, **b** glycerol, **c** glucose, and **d** ethanol concentrations versus time

### Growth characteristics

**Fig. 3:**
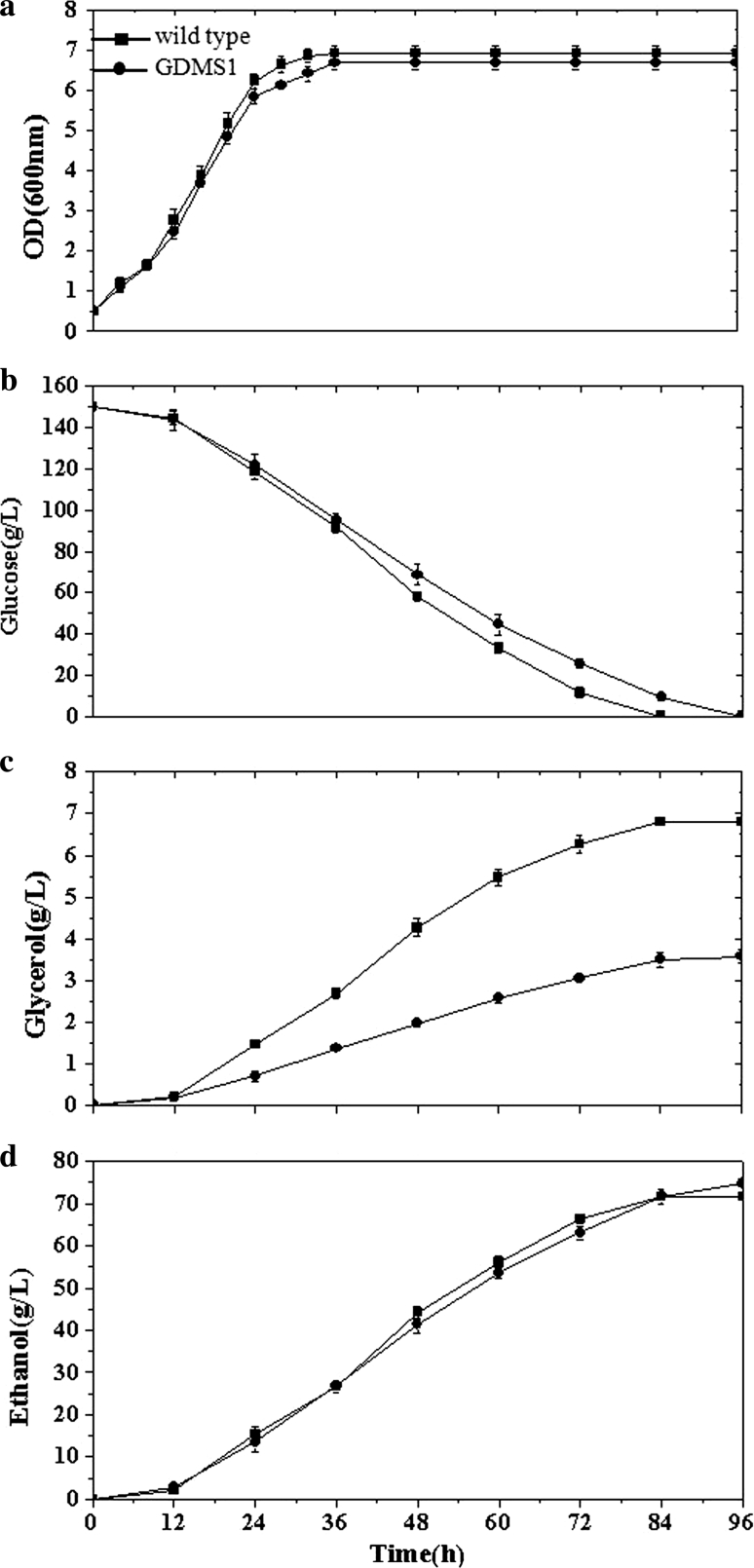
Comparison of recombinant strain *S. cerevisiae* GDMS1 and wild type when acetic acid was added prior to inoculation: **a** OD_600_, optical density at 600 nm, **b** glycerol, **c** glucose, and **d** ethanol concentrations versus time

The effects of the introduced genetic changes on the cellular physiology of *S. cerevisiae* CICIMY0086 were studied under anaerobic growth conditions on 15 % glucose. Wild-type strain grown under similar conditions was used as the control. The recombinant strains expressing the heterologous genes did not show any significant decline in μ_max_ compare to the wild type (Fig. 2a). However, when 2 g acetic acid l^−1^ was added to the medium prior to inoculation, the wild type and recombinant strain GDMS1 both showed slightly lower growth rates (Fig. 3a). In addition, 84 h was required for the wild type and the recombinant strains to consume all the glucose. When acetic acid was added to the medium, the fermentation period of the GDMS1 was prolonged by approximately 12 h. All recombinant strains yielded a similar concentration of biomass compared to the wild type at the end of fermentation (Table 3).

**Table 3:** Comparison of recombinant strain *S. cerevisiae* GDS1, *S. cerevisiae* POS1, *S. cerevisiae* GDMS1, and wild type during anaerobic batch growth on 15 % glucose

Yeast strain	Ethanol (g l^−1^)	Yield on glucose	
		Biomass (g g^−1^)	Glycerol (g g^−1^)
Wild type	71.3 ± 1.1	0.042 ± 0.003	0.047 ± 0.003
GDS1	73.4 ± 1.3	0.041 ± 0.003	0.035 ± 0.002
POS1	74.0 ± 1.4	0.042 ± 0.003	0.040 ± 0.002
GDMS1	74.5 ± 1.0	0.041 ± 0.002	0.029 ± 0.001

### Analysis of fermentation products

HPLC analysis of the fermentation products revealed that co-expression of *GCY1* and *DAK1* increased the ethanol yield for strain *S. cerevisiae* GDS1 by 2.9 % compared with that of the wild type (relative to the amount of substrate consumed), while glycerol formation dropped by 24.9 % (relative to the amount of substrate consumed) during anaerobic batch fermentations (Fig. 2; Table 3). Enhancing the dihydroxyacetone pathway by co-expressing *GCY1* and *DAK1*, encoding glycerol dehydrogenase and dihydroxyacetone kinase, respectively, redirected the carbon flow to the glycolytic intermediate dihydroxyacetone phosphate, resulting in greater overall ethanol production.

The strain POS1 expressing *POS5* generated a 3.8 % increase in ethanol yield and 15.2 % decrease in glycerol production in batch culture compared to the wild type. Expressing the *mhpF* gene in the recombinant strain GDMS1 resulted in a 4.5 % increase in ethanol yield and 38 % decrease in glycerol production compared to the wild type, without the addition of acetic acid during anaerobic batch fermentations (Fig. 2; Table 3). When acetic acid was added prior to inoculation, GDMS1 showed a 5.5 % increase in ethanol yield and 48 % decrease in glycerol yield compared to that of the wild type (Fig. 3; Table 4). Supplementation with acetic acid reduced the biomass yield of the GDMS1 strain compared with no supplementation.

**Table 4:** Comparison of recombinant strain *S. cerevisiae* GDMS1 and wild type during anaerobic batch growth on 15 % glucose supplemented with acetate before inoculation

Yeast strain	Ethanol (g l^−1^)	Yield on (glucose + acetate)	
		Biomass (g g^−1^)	Glycerol (g g^−1^)
Wild type	71.0 ± 1.0	0.036 ± 0.001	0.045 ± 0.002
GDMS1	74.9 ± 1.4	0.034 ± 0.002	0.024 ± 0.002

### Analysis of intracellular NADH

Intracellular NADH was extracted according to the method previously described [12], after which the extracts were analyzed in an enzymatic cycling system. Intracellular NADH in wild-type strain was determined to be 0.0152 mmol/g-DCW (dry cell weight). Levels of intracellular NADH in GDS1, GAS1, MHS1, POS1, and FRS1 were 0.0273, 0.0132, 0.0131, 0.0138, and 0.0325 mmol/g-DCW, respectively (Table 5). Expressing *mhpF* in GDS1 (GDMS1) yielded an intracellular NADH concentration of 0.0178 mmol/g-DCW, lower than the 0.0273 mmol/g-DCW measured in the recombinant strain *S. cerevisiae* GDS1, but higher than that of the parent strain (Table 5).

**Table 5:** Intracellular NADH content

Strain	NADH^a^ concentration (mmol/l, ±SD)	NADH^a^ (mmol/g-DCW, ±SD)
*S. cerevisiae*	0.0543 ± 0.0001	0.0152 ± 0.0001
*S. cerevisiae* GDS1	0.0862 ± 0.0002	0.0273 ± 0.0002
*S. cerevisiae* GAS1	0.0395 ± 0.0001	0.0132 ± 0.0001
*S. cerevisiae* POS1	0.0441 ± 0.0002	0.0138 ± 0.0002
*S. cerevisiae* MHS1	0.0292 ± 0.0001	0.0131 ± 0.0001
*S. cerevisiae* FRS1	0.0737 ± 0.0001	0.0325 ± 0.0001
*S. cerevisiae* GDMS1	0.0646 ± 0.0002	0.0178 ± 0.0002

^a^Triplicate experiments

## Discussion

Glycerol synthesis plays a critical physiological role in maintaining the osmotic constancy and redox balance of yeast cells under anaerobic conditions [4, 16]. Additional glycerol is used to synthesize the cellular membrane. Simply interrupting glycerol synthesis to improve ethanol yield has proved unsuccessful, with the growth and product formation of such engineered strains being severely impaired [3]. Since completely eliminating the production of glycerol is unrealistic, a better alternative is to generate glycerol at a much lower rate while ensuring the engineered yeast remains true to its wild-type phenotype.

The present study is the first to couple glycerol degradation with ethanol formation, to the best of our knowledge. By overexpressing *GCY1* and *DAK1* in *S. cerevisiae*, glycerol was converted to glycolytic intermediates and then ethanol. However, overexpressing *GCY1* and *DAK1* in *S. cerevisiae* increased the intracellular NADH content. Under aerobic conditions, surplus NADH formed in metabolic reactions is reoxidized to NAD^+^ by mitochondrial respiration, whereas under anaerobic conditions, glycerol formation by yeast is essential to reoxidize NADH [2, 23]. Thus, the increase in ethanol yield and decrease in glycerol yield were not as high as expected.

Ethanol yields may thus be further boosted by preventing the additional formation of NADH. In our previous work [24], targeting cofactor regulation decreased glycerol production in *S. cerevisiae*. Three different genes, *gapN*, *frdA*, and *mhpF*, were overexpressed in *S. cerevisiae* separately. Glycerol in the yeast expressing *gapN* decreased by 23.4 %, with only a 3.5 % increase in ethanol yield compared with the wild type. Recombinant strains *S. cerevisiae* MHS1 expressing *mphF* and *S. cerevisiae* FRS1 expressing *frdA* needed acetic acid or fumarate to be added as electron acceptors, respectively. When acetic acid was added prior to inoculation, *S. cerevisiae* MHS1 generated a 4.3 % increase in ethanol yield and 40 % decrease in glycerol yield compared to the wild type, whereas the strain *S. cerevisiae* FRS1 expressing *frdA* boosted levels of glycerol [24]. Thus, we chose to overexpress *mphF* in the recombinant strain GDS1 to reduce NADH formation. Interestingly, the intracellular NADH concentration for the recombinant strain GDMS1 was found to be higher than that of the parent strain. This is inconsistent with previous reports whereby introducing a new linear pathway was found to relieve the toxic effect of surplus NADH [20]. However, a further reduction in glycerol production and increase in ethanol yield were observed in the strain GDMS1 when acetic acid was added prior to inoculation. This may be because the glycerol degradation pathway was more efficient than the *gapN*-catalyzed reaction. Thus, more NADH was generated than reduced.

The gene *POS5* encoding NADH kinase was also overexpressed in *S. cerevisiae* for comparison. The resultant strain POS1 exhibited a 3.8 % increase in ethanol yield and 15.2 % decrease in glycerol production in batch culture compared with the wild type. Even though the increase in ethanol yield was lower than the previously reported 8 % for *S. cerevisiae* overexpressing NADH kinase [10], our results nevertheless demonstrate that such a strategy can work well in industrial strains such as *S. cerevisiae*.

Further research is needed to explain the observed increases in ethanol yield and decreases in glycerol production. In addition, the strategy needs to be refined to reduce the intracellular concentration of NADH, for example by enhancing the dihydroxyacetone route in a mutant deletion of *GPD2* while at the same time introducing a more efficient NADH-dependent pathway to maintain the redox balance.

## Electronic supplementary material

Below is the link to the electronic supplementary material.

Supplementary Fig 1. Schematic summary of the construction of the plasmid pMGKR-*GCY1*-*DAK1* in the study. *PGK1* promoter, *S. cerevisiae* glyceraldehyde 3-phosphate dehydrogenase gene promoter; *Kan,* kanamycin resistance gene from pPIC9 K which confers resistance to geneticin in *S. cerevisiae* and kanamycin resistance in *E. coli* (JPG 60 kb)
