# Supplementary figures and images for: Engineering of the glycerol decomposition pathway and cofactor regulation in an industrial yeast improves ethanol production
Source: J Ind Microbiol Biotechnol. 2013 Jul 30;40(10):1153–60. doi: 10.1007/s10295-013-1311-5 (PMC3769588; doi:10.1007/s10295-013-1311-5)

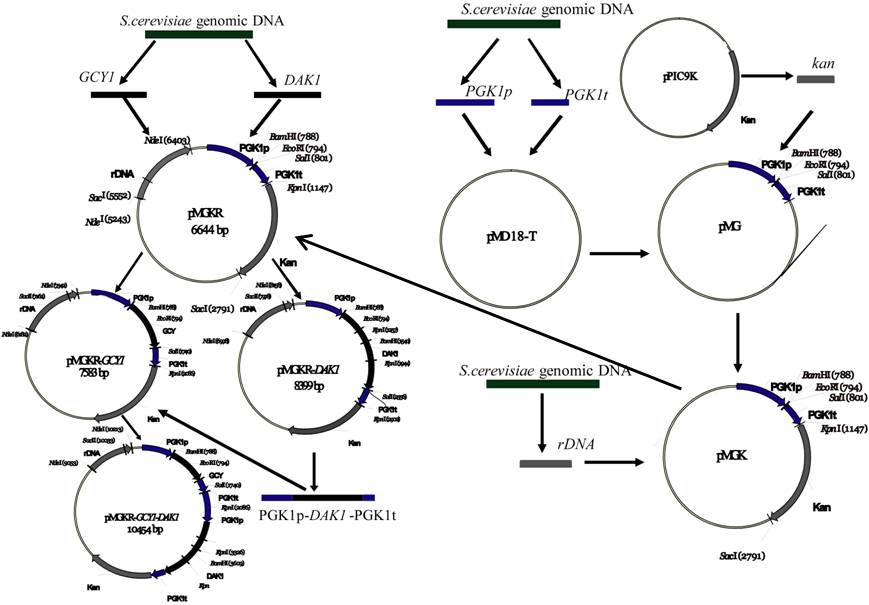

Supplement: Supplementary file 1 — Supplementary Fig 1. Schematic summary of the construction of the plasmid pMGKR-GCY1-DAK1 in the study. PGK1 promoter, S. cerevisiae glyceraldehyde 3-phosphate dehydrogenase gene promoter; Kan, kanamycin resistance gene from pPIC9 K which confers resistance to geneticin in S. cerevisiae and kanamycin resistance in E. coli (JPG 60 kb) [file 10295_2013_1311_MOESM1_ESM.jpg]
